# Maximizing the Scope of Practice among Mental Health Nurses: Regional, Educational, and Setting-Based Differences

**DOI:** 10.1192/j.eurpsy.2026.11660

**Published:** 2026-07-31

**Authors:** V. Rozani, A. Romem

**Affiliations:** 1 Nursing, Tel Aviv Universty, Tel Aviv; 2 Nursing, The Jerusalem College of Technology - Lev Academic Center, Jerusalem, Israel

## Abstract

**Introduction:**

Mental health nurses play a critical role in delivering complex, person-centered care across hospital and community settings. Globally, there is a growing emphasis on maximizing nurses’ scope of practice to enhance care quality, workforce efficiency, and professional satisfaction. However, in Israel, limited data exist on the extent to which mental health nurses apply their advanced competencies in clinical settings.

**Objectives:**

This study aimed to assess the extent to which mental health nurses in Israel utilize their full scope of practice and to identify factors that facilitate or hinder this utilization.

**Methods:**

A cross-sectional survey was conducted among a convenience sample of 282 registered nurses who had completed post-basic certification in mental health nursing. Participants completed a structured questionnaire addressing socio-demographic and occupational characteristics, implementation of scope-of-practice activities, and professional autonomy and job satisfaction.

**Results:**

The sample included a relatively high proportion of male nurses (35.5%) compared to other nursing specialties. The mean age was 44.1 years, and 51.4% held a master’s degree. Scope of practice varied significantly by region, with nurses in the central district reporting the highest levels of role implementation (p<.001). Community-based nurses reported significantly greater professional autonomy, readiness, and empowerment compared to their hospital-based counterparts (p<.01). In addition, nurses with higher educational attainment (MA/PhD) and greater seniority reported significantly higher scores across multiple domains of professional actualization and valuation (p<.05 to p<.001), suggesting that advanced qualifications and experience support broader scope utilization.

**Conclusions:**

Mental health nurses in community settings reported greater professional autonomy, readiness, and empowerment compared to those in hospitals, suggesting broader role utilization outside institutional environments. Nurses with higher education and seniority demonstrated greater actualization and valuation of their roles, indicating that advanced training and experience support expanded scope of practice. Regional disparities, particularly higher scores in the central district, highlight potential inequities in access to supportive infrastructure and professional opportunities.

**Disclosure of Interest:**

None Declared

